# Are sex differences in self‐estimated intelligence an elusive phenomenon? Exploring the role of working memory, creativity, and other psychological correlates in young and older adults

**DOI:** 10.1002/brb3.2857

**Published:** 2023-01-26

**Authors:** Vaitsa Giannouli

**Affiliations:** ^1^ Department of Social and Behavioral Sciences European University Cyprus Nicosia Cyprus; ^2^ Department of Psychology University of Western Macedonia Florina Greece; ^3^ Department of Educational and Social Policy University of Macedonia Thessaloniki Greece

**Keywords:** age, attitudes, creativity, intelligence, self‐estimated, sex differences, working memory

## Abstract

**Background:**

Although there is research examining the demographic predictors of self‐estimated intelligence (SEI) in young adults, so far SEI in old age is little investigated. This study aims to examine the influence of additional variables such as self‐estimated emotional intelligence (SEEQ), physical attractiveness, health, general optimism, religiousness, and working memory (WM) on SEI both in young and older adults.

**Methods:**

A total of 159 young (90 women, *M*_age_ = 28.77, *SD* = 8.83) and 152 older adults (93 women, *M*_age_ = 71.92, *SD* = 6.84) completed a measure of SEI as well as questions regarding the abovementioned variables. Given that WM is considered a very strong predictor of intelligence, neuropsychological assessment included the measurement of WM and phonologically cued semantic retrieval–verbal storage and processing in WM, as assessed by the Digit Span Forward and Verbal Fluency Task. The visual storage in WM was assessed with a variation of the Visual Patterns Test, and the visual storage and processing in WM with the Corsi blocks task (backward). Positive and Negative Affect Schedule (PANAS‐X) was also administered as a possible influence on cognitive performance and SEI.

**Results:**

Young males rated their intelligence quotient (IQ) and emotional quotient (EQ) higher than young females. This was not confirmed for older adults, for which surprisingly the reversed pattern was found. Older women reported higher IQ and EQ than older men. Correlations showed for all participants that the higher they rated their IQ, the higher their ratings of EQ, physical attractiveness, health, and religiousness. No significant correlations between objective tests regarding WM and SEI were found, supporting SEI overestimations. Age, sex, physical attractiveness, and SEEQ were significant predictors of SEI.

**Discussion:**

For the first time, a reverse sex difference across age groups in SEI is found. Implications for individuals and healthcare professionals involved in assessment are suggested.

## Graphical abstract

This research measures self‐estimated intelligence along with other variables for the first time in an extended sample of young and older adults, with an emphasis on old age, as this is an age group so far little investigated. The novelty of this study lies not only in the comparisons of young and older participants, and the cultural context (first study in Greece), but also in the neuropsychological assessment that was performed for the whole sample, as well as the inclusion of relevant correlates of self‐estimated intelligence found in previous published articles.

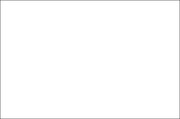

## 1 INTRODUCTION

Self‐estimated intelligence (SEI) refers to the self‐estimates of people regarding their intellectual abilities (Hollig & Preckel, 2005). Although SEI has attracted the interest of researchers as mentioned in numerous reviews (Freund & Kasten, 2012; Heck et al., 2018; Kaufman, 2012, 2019; Neto, 2019; Syzmanowicz & Furnham, 2011; von Stumm, 2014), most studies find weak to moderate correlations between self‐estimated and tested intelligence aspects (Hollig & Preckel, 2005), while other researchers support that there is a reasonably accurate estimation of intelligence score, with correlations between estimates and actual scores ranging between Pearson's *r* = .2 and *r* = .4 (Furnham, 2001). A possible explanation behind this may be the “self‐esteem bias” (Felson, 1981), which can be described as the tendency for people to evaluate themselves in a way that is consistent with their general self‐esteem. Thus, someone who is high in self‐esteem will tend to see themselves as brighter and more capable than someone lacking in self‐esteem even if they are indeed demonstrating a higher objective intelligence (Reilly et al., 2022; Syzmanowicz & Furnham, 2011).

The cross‐cultural perspective reveals additionally a significant sex difference, with young adult males reporting higher estimates than females (Furnham & Grover, 2020) in a relevant cross‐cultural research in Australia, Austria, Brazil, France, Iran, Israel, Malaysia, New Zealand, South Africa, Spain, Turkey, the United Kingdom, and the United States (von Stumm et al., 2009). Although there are additional data that confirm the sex difference in countries, such as Austria (Stieger et al., 2010), Spain (Perez et al., 2010), Switzerland (Proyer, 2011), Russia (Kornilova &, Novikova, 2012), Wales (Workman, 2004), Pakistan (Shahzada et al., 2014), Tanzania (Dixon et al., 2016), as well as Uganda (Furnham & Baguma, 1999), no study so far has compared sex differences in SEI in Greece. A number of factors have been proposed as predictors of SEI, such as gender, prior experience with the intelligence testing, and extraversion (Zhang & Gong, 2001). This “male hubris, female humility” (MHFH) problem (Furnham et al., 2001) is also found in adolescents (Neto et al., 2009), but cannot be supported by objective differences in general intelligence, as gender differences are only found for specific cognitive abilities and more specifically for verbal and visual–spatial tasks rather than psychometric intelligence (Halpern et al., 2011). A possible mechanism explaining MHFH could be that individuals in general are motivated to keep their self‐concept consistent with traditional gender norms of their biological sex, which is characterized as appropriate for or of one sex rather than the other (Maccoby, 1990; Martin & Ruble, 2004), and these implicit beliefs about gender and intellectuality may influence self‐reports in the form of higher estimates and overconfidence with regard to their own intelligence if they are males and lower estimates/lack of confidence if they are females (Reilly & Mulhern, 1995). Additionally, research investigating personality supports that sex and personality effects are largely independent (Stieger et al., 2010). Besides gender, another moderator is the level of education regarding self‐estimates of specific aspects of intelligence (Rammstedt & Rammsayer, 2002) as evidence supports small education effects, with better educated people tending to give higher SEI (Furnham et al., 2002; Rammstedt & Rammsayer, 2002).

Self‐estimated emotional intelligence (SEEQ) has been also investigated (Giannouli, 2017a). Overall, psychometric intelligence is considered to be a primarily masculine attribute in contrast with emotional intelligence (EI), which is perceived as a primarily feminine attribute (Petrides et al., 2004; 2010). Although it is supported that males estimate their spatial and mathematical intelligence higher than females, they tend to estimate lower their EI than females (Furnham, 2017). These differences between males and females regarding EI have been found to be reverse in another study, as males believe that they have higher trait total EI than females (Petrides & Furnham, 2000).

Creativity on the other hand is a novel and still little investigated concept (Giannouli, 2018), regarding SEI research. Males again have been found to give higher estimates than females in SEI and creativity (Furnham et al., 2005). SEI and creativity, as well as the Big Five personality traits, seem to predict psychometric intelligence (Furnham et al., 2005).

Although attempts have been made for the development of reliable and valid self‐report measures of cognitive abilities in older individuals (Herreen & Zajac, 2018), so far objective cognitive abilities have not been thoroughly examined along with SEI in large samples not only of young but also older adults. Preliminary research has shown discrepancies of objective cognitive abilities and self‐reports in older patients diagnosed with cognitive deficits (Giannouli & Tsolaki, 2015). Thus, one more relevant variable to consider in objective cognitive testing should be working memory (WM), which is considered not as isomorphic with intelligence factors, but as a very strong predictor (Oberauer et al., 2005). Finally, positive and negative affects at the time of neuropsychological testing were also included as neglected possible influences on cognitive performance as well as on SEI (Giannouli, 2017b), based on findings supporting that negative affect (e.g., taking the form of stress) influences both intelligence quotient (IQ) and emotional quotient (EQ) (Jung et al., 2019), but also on findings supporting that state negative affect influences self‐estimates of cognitive performance especially in older adults (even those without a diagnosis of depression [Giannouli & Tsolaki, 2022a]).

Furthermore, although there are no reported age differences in SEI, as so far males of all ages have been found to estimate 5−15 IQ points higher than females in Western settings (Furnham, 2017), for SEEQ increased EI and related perceptions are found in old age compared to younger adults (Chen et al., 2016; Sharma, 2017). Regarding creativity there is an open debate on the adverse effects of age in later life (Lindauer, 2003; Nakamura & Csikszentmihalyi, 2003), as age differences have been found to be the disadvantage of older adults in some studies (e.g., Abra, 1989; Ruth & Birren, 1985), but there are contrasting findings showing that creativity as well as perceptions of creativity do not decline in the later years of life in other studies (e.g., Fisher & Specht, 1999; Lorenzen‐Huber, 1991). It is worth mentioning that there is a plethora of studies about sex and age differences in WM variables, supporting a male advantage in visual–spatial WM (Voyer et al., 2017), but for verbal WM a female advantage is reported (Voyer et al., 2021).

Estimations of physical attractiveness and physical health have been examined in a recent study in relation to SEI, showing a high positive correlation, something that means that those who rate their IQ highly also believe that they are more attractive and healthy (Furnham & Grover, 2020). Men's and women's ratings of self‐perception of attractiveness do not significantly differ (Talbot, 2012), but there is contrasting evidence regarding physical health, as it has been found that women tend to evaluate their health as “poor,” and on average report more symptoms than men (Anson et al., 1993), while other studies support that regardless of disease burden, no such sex differences exist (Sood et al., 2019). For religiousness, only one research has examined its impact on SEI revealing a significant positive correlation (Furnham & Grover, 2020), but there are no data in settings where the dominant religion is other than protestant or catholic, as is the case of Greece. Findings in the Greek Orthodox religious and cultural setting support moderate positive correlations between self‐esteem and perceived religiousness (Giannoulis & Giannouli, 2020a) as well as between EI and religiousness (Giannouli & Giannoulis, 2020). Relevant prior research supports no sex differences in religiousness between healthy Greek younger and older adults (Giannoulis & Giannouli, 2020b).

Based on the above, this study aims to extend previous research (Furnham & Grover, 2020) by examining SEI, and its hypothesized correlates such as SEEQ, ratings of physical attractiveness, health, optimism, and religiousness, along with estimations of creativity, and objective cognitive functions, such as WM, for the first time in Greece in a sample of participants, with varying ages. Thus, this study is addressing two important research questions, namely, (1) are the frequently reported sex differences in SEI present in samples of older adults, and (2) which are the psychological correlates in old age compared to younger adults? The choice of different age groups was based on the fact that as all previous studies mainly focused on young adults (e.g., undergraduate students [Zhang & Gong, 2001] and/or young adults [Furnham & Grover, 2020]). In addition, since cultural characteristics may influence not only perceptions, attitudes, and beliefs, but also cognitive performance (Lezak et al., 2012), this is the first study to present data from Greece.

## 2 MATERIALS AND METHODS

### 2.1 Participants

Overall, there were 311 adults, of which 128 were male and 183 females. They were, on average, 55.86 years old (*SD* = 23.00). The average years of formal education for the whole sample were 11.08 (*SD* = 4.39). More specifically, 159 were young adults (90 women; *M*_age_ = 34.77, *SD* = 8.83; *M*_education_ = 14.35, *SD* = 1.39) and 152 were community dwelling older adults (93 women; *M*_age_ = 77.92, *SD* = 6.84; *M*_education_ = 7.65, *SD* = 3.79). The two age groups were similar in terms of gender distribution (*χ* ^2^(1) = .673, *p* = .422). Participants were recruited from a pool of volunteers that had participated in a previous study of cognitive ageing and did not receive financial compensation (Giannouli, 2018). The choice of 65 years as a cutoff for grouping younger and older adults (defined here as persons aged ≥65 years) was based on the fact that most studies worldwide have accepted the chronological age of 65 years as a definition of “elderly” or older person. Although some participants from the group of older adults (aged over 65) had been following medication related to cardiovascular diseases, they had no official diagnosis of a cognitive deficit/neurocognitive disorder, and had scored above 27 points on the Greek version of the Mini Mental State Examination (MMSE), which is used in order to exclude neurocognitive disorders. This test was included as previous research supports overestimation of cognitive abilities in dementias by the patients themselves (Giannouli & Tsolaki, 2021).

Exclusion criteria for both groups of younger and older participants were a history of psychiatric, neurological, or substance abuse dependence, a history of head injury or any other medical condition (including significant perceptual deficits such as visual and/or hearing impairments not corrected sufficiently by aids) that might affect neuropsychological performance, and non‐native speakers of the Greek language.

### 2.2 Questionnaire of estimates

Participants were asked to estimate, on a scale from 0 to 100 as in the original study by Furnham and Grover (2020), their overall intelligence (Male = 77.92, *SD* = 13.01; Female = 74.92, *SD* = 13.30; *t*(309) = 2.016, *p* = .04), EI (Male = 76.79, *SD* = 12.71; Female = 77.06, *SD* = 10.96; *t*(309) = 0.199, *p* = .842), physical health (Male = 75.01, *SD* = 14.24; Female = 77.02, *SD* = 38.40; *t*(309) = 0.564, *p* = .573), and physical attractiveness (Male = 76.73, *SD* = 12.93; Female = 76.95, *SD* = 10.93; *t*(309) = 0.163, *p* = .870). Using a 9‐point scale from 1 = *Not at all* to 9 = *Very*, they rated to what extent they were an optimist (Male = 3.21, *SD* = 1.08; Female = 3.32, *SD* = 1.12; *t*(309) = 0.873, *p* = .383) and how religious they were (Male = 4.03, *SD* = 1.71; Female = 4.13, *SD* = 1.81; *t*(309) = 0.489, *p* = .625).

### 2.3 Neuropsychological tests

Different aspects of WM were assessed with four standardized neuropsychological tests for which there are research data of their appropriateness for the Greek population, namely, Visual Patterns Test, Corsi blocks task (backward condition), Digit Span Forward, and Verbal Fluency Task. Although studies have demonstrated inconsistent evidence concerning the characterization of these tests as appropriate for the assessment of WM or short‐term memory (STM) (Engle et al., 1999; Shao et al., 2014; Shipstead et al., 2016), research on the other hand supports that spatial WM is assessed by the Corsi blocks task (Higo et al., 2014; Milner, 1971), WM verbal storage ability is reflected in digit forward span (Kremen et al., 2008), WM is needed for both letter and category fluency (Rende et al., 2002), and Visual Patterns Test is used in neuropsychological assessment protocols of WM (Brown et al., 2012; Kouvatsou et al., 2019).

#### 2.3.1 Visual storage in WM

##### Visual Patterns Test

A variation of the Visual Patterns Test was administered. The participants were presented with cards that had on them combinations of checkerboard patterns, which have been constructed in such a way that it is not easy to encode the patterns verbally. A visual pattern is produced by filling in half of the squares in a presented grid, so some of the squares are black and the others white (Della Salla et al., 1997). The participants had to reproduce the black squares they saw in the presented cards by indicating with pencil on the answer sheet grid the respective squares. The exact instructions to the participants were as follows: “This task tests the memory for visual images. You are going to see a pattern like this one (showing a stimulus card) and I will ask you to recall it by drawing in these empty grids (showing the answering sheet). You are going to look at the pattern for a short period of time. For that reason, you should concentrate and look carefully in order to recall the pattern immediately afterward. As soon as I cover the pattern you should start drawing. The patterns are easier in the beginning and become more difficult later on.”

#### 2.3.2 Visual storage and processing in WM

##### Corsi blocks task (backward condition)

The Corsi task assesses visuospatial WM (Milner, 1971). It involves mimicking a researcher as he/she taps a sequence of up to nine identical spatially separated blocks. The sequence starts out simple, usually using two blocks, but becomes more complex until the subject's performance suffers (Berch et al., 1998). Each stimulus item comprised a tapping pattern performed by the examiner who pointed sequentially to a subgroup of the nine blocks. Participants were asked to copy the tapping pattern, which was indicated by the examiner in a backward manner. The sequence complexity increased from one tap to nine taps at the highest level. At each level of complexity, participants were asked to reproduce six sequences. The exact instructions were as follows: “Look carefully at this wooden template. It has nine cubes pegged on it. I will touch two of the cubes and I would ask you to look at this carefully in order to touch the same cubes right after me in reverse order. Are you ready?”

#### 2.3.3 Verbal storage in WM

##### Digit Span Forward

The Digit Span Forward from the Wechsler Adult Intelligence Scale‐III (Wechsler, 1997) consists of a series of one‐digit numbers from 2 to 14 digits, which are read in a rate of one digit per second. The participants had to write down their answers for two sets of numbers per digit length. In the statistical analyses in order to claim that a maximum span was achieved, at least one of the two trials had to be correct, and scoring by the examiner ended when a participant was incorrect on two trials of the same length.

#### 2.3.4 Verbal storage and processing in WM

##### Verbal Fluency Task

The Verbal Fluency Task requires the production in written form of as many words as possible beginning with a specified letter from the Greek alphabet (e.g., participants were asked to generate as many different words as possible beginning with the Greek letters chi, sigma, alpha, pi, excluding proper nouns, and variations of the same word) (phonemic fluency) (Kosmidis et al., 2004). The phonological condition of the word fluency test imposes strong demands on executive functioning and on phonological WM (Giannouli, 2018). In this testing, the participants were given a timeframe of 1 min. In the analyses, only the number of correct responses was used.

#### 2.3.5 State affect

##### Positive and Negative Affect Schedule

Positive and Negative Affect Schedule (PANAS‐X) is a brief self‐report questionnaire that consists of two 10‐item scales and can measure both positive and negative affects. Each item is rated on a Likert scale of 1 (*Not at all*) to 5 (*Very much*) (Watson & Clark, 1999). The Cronbach's alpha coefficient was acceptable for both the negative affect items (*α* = .769) and for the positive affect items (*α* = .763).

#### 2.3.6 Creativity

##### Creative Attitudes and Values, section of Runco's Creativity Assessment Battery

The Creative Attitudes and Values measurement, which is part of the Runco Creativity Assessment Battery (rCAB^©^; Runco, 2012), was used for measuring creativity after permission was obtained by the first author from the creator. The Creative Attitudes and Values tool consists of 25, 5‐point Likert scale items. Out of these 25 items, 15 and 10 were indicative and contraindicative items, respectively. Contraindicative items were reverse coded and were used along with the indicative ones. The Cronbach's alpha for this sample was acceptable (*α* = .705). The purpose of the Creative Attitudes and Values questions was to try and assess a person's attitudes and values about creativity, their tendencies to act creatively, and their affinity for creativity. Attitudes can greatly influence the probability of thinking and feeling in a creative fashion. Values concerning creativity are incorporated in the score as well to indicate how much a person appreciates divergence, originality, autonomy, and so forth. Attitudes and values are both predictive of actual behavior (Acar & Runco, 2014).

### 2.4 Procedure

Participants were recruited from the first phase of a previous large‐scale experiment in which other cognitive domains had been tested (Giannouli, 2018). They were told their anonymous results would be used for analysis, while there was no monetary or other explicit reinforcement/incentive for getting tested. Therefore, all participants had test experience. Completing the demographics questionnaire, the questionnaire of estimates, and the neuropsychological testing took, on average, 34 min for younger adults and 58 min for older adults.

### 2.5 Data analyses

The statistical analyses were performed using the IBM SPSS (Statistical Package for Social Science) Statistics software, Version 24.0. Means and standard deviations were computed. To examine the relationships between SEI and previously mentioned related variables in the scientific literature, such as SEEQ, ratings of physical attractiveness, health, optimism, and religiousness, along with estimations of creativity, and objective cognitive functions focusing on WM as measured by the Digit Span Forward, Verbal Fluency Task, Visual Patterns Test, and the Corsi blocks task (backward condition) as well as affect measured by PANAS‐X, Pearson correlations were calculated. The bivariate Pearson correlation measures the strength and direction of linear relationships between pairs of continuous variables. In addition, two‐way analysis of variance (ANOVA) was applied for an age group × sex interaction and the abovementioned variables, and partial eta squared as a measure of effect size is presented. Bonferroni post hoc analysis tests were applied to examine significant differences between means. Linear regression (Stepwise Method) was conducted with SEI as dependent variable and the relevant correlates as independent variables; *p*‐values <.05 were considered significant.

## 3 RESULTS

Means and standard deviations are presented for all administered questions and tests for the total sample (see Table 1).

**TABLE 1:** Bivariate correlations between sex, neuropsychological tests, and the other study variables in the group of younger adults

Measure	1	2	3	4	5	6	7	8	9	10	11	12	13	14
1. Sex^a^	–	–.513^*^	–.328^**^	–.313^**^	–.305^**^	.052	–.005	.025	–.002	.153	–.033	.015	.025	–.010
2. SEI		–	.751^**^	.734^**^	.711^**^	–.048	.075	–.164^*^	.055	–.162^*^	.084	.043	–.007	–.033
3. SEEQ			–	.982^**^	.951^**^	–.165^*^	.180^*^	–.090	.003	–.056	.146	.034	.020	–.112
4. Physical attractiveness				–	.946^**^	–.172^*^	.166^*^	–.074	–.033	–.037	.149	.039	.016	–.087
5. Health ratings					–	–.157^*^	.188^*^	–.099	–.008	–.036	.125	.033	.039	–.115
6. Optimism						–	–.157^*^	.134	–.013	–.059	–.257^**^	–.222^**^	–.127	.061
7. Religiousness							–	.095	.101	.066	.084	–.025	.118	–.022
8. Creative Attitudes and Values								–	–.032	–.059	.045	–.091	.056	.092
9. Positive PANAS									–	.058	–.044	.146	–.073	.115
10. Negative PANAS										–	.043	–.098	.025	–.180^*^
11. Digit Span Forward											–	–.043	.175^*^	–.58
12. Verbal Fluency Task												–	.006	.283^**^
13. Corsi blocks (backward condition)													–	.278^**^
14. Visual Patterns Test														–

^a^ Dummy coded variable; 0 = male, 1 = female.

^*^ *p* < .05; **p < .01.

Pearson's *r* correlation coefficients were calculated for the abovementioned variables (see Table 2 for the variables in the total sample, as well as Tables 1 and 2 for separate correlations in younger and older age groups).

**TABLE 2:** Bivariate correlations between sex, neuropsychological tests, and the other study variables in the group of older adults

Measure	1	2	3	4	5	6	7	8	9	10	11	12	13	14
1. Sex^a^	–	.350^**^	.432^**^	.326^**^	.201^*^	.060	.056	–.083	–.041	.186*	–.034	–.074	–.044	–.006
2. SEI		–	.809^**^	.956^**^	.556^**^	.051	.192^*^	.010	–.064	–.102	.179	.027	.050	–.150
3. SEEQ			–	.829^**^	.472^**^	.073	.160^*^	–.151	–.099	.038	.099	.096	–.040	–.203*
4. Physical attractiveness				–	.599^**^	.028	.155	–.056	–.031	–.079	.204^*^	.012	.033	–.155
5. Health ratings					–	–.004	.249^**^	–.035	–.063	–.001	.020	.161*	–.019	–.054
6. Optimism						–	–.057	.162	.077	.010	.088	–.099	.061	.157
7. Religiousness							–	.131	–.116	–.119	.017	.300^**^	.095	–.239^**^
8. Creative Attitudes and Values								–	–.001	–.247	.155	–.018	.190*	.133
9. Positive PANAS									–	.300*	.076	–.028	.042	.151
10. Negative PANAS										–	–.086	.169*	–.131	–.037
11. Digit Span Forward											–	.010	.224^**^	.108
12. Verbal Fluency Task												–	.042	–.141
13. Corsi blocks (backward condition)													–	.189^**^
14. Visual Patterns Test														–

^a^ Dummy coded variable; 0 = male, 1 = female.

^*^ *p* < .05; ^**^ *p* < .01.

Pearson correlations for the whole sample revealed a number of statistically significant correlations. Namely, SEI correlated positively with age (*r* = .144, *p* = .011), SEEQ (*r* = .847, *p* < .001), physical attractiveness (*r* = .834, *p* < .001), health ratings (*r* = .336, *p* < .001), and religiousness (*r* = .131, *p* < .001). Only one statistically negative significant correlation was found for SEI and the negative affect subscale of PANAS (*r* = –.142, *p* = .014).

When correlations were performed separately for the group of younger adults, statistically significant positive correlations were found between SEI and SEEQ, physical attractiveness, and health ratings, while statistically significant negative correlations were found between SEI and creativity as well as negative affect (see Table 1).

For the group of older adults, similar statistically significant positive correlations were found between SEI and SEEQ, physical attractiveness, and health ratings, with the addition of religiousness and performance on Digit Span Forward. No statistically significant negative correlations were found (see Table 2).

A two‐way ANOVA with two independent variables (age group [young and older participants over 65 years] and sex [males and females]) revealed that for SEI there was no significant main influence of age group (*F*(1/307) = 0.943, *p* = .332, *η*_p_^2^ = .003), but there was a significant influence of sex with a small effect size (*F*(1/307) = 4.129, *p* = .043, *η*_p_^2^ = .013) with males in general demonstrating higher SEI (*M* = 77.99, *SD* = 13.01) compared to females (*M* = 74.92, *SD* = 13.30), as well as an interaction of age group × sex that is of great interest with a large effect size (*F*(1/307) = 72.389, *p* < .001, *η*_p_^2^ = .191) with young females showing the lowest SEI, followed by old males, old females, and young males. In this case of 2 × 2 ANOVA, Estimated Marginal Means were used, given that post hoc tests do not apply (Table 3).

**TABLE 3:** Descriptive statistics regarding SEI in young and older adult groups according to sex

Age group	Sex	Mean	*SD*	95% CI		*N*
Young	Males	82.72	14.27	79.92	85.52	69
	Females	68.36	9.91	65.91	70.81	90
Old	Males	72.45	8.61	69.43	75.48	59
	Females	81.27	13.12	78.87	83.68	93

A two‐way ANOVA with two independent variables (age group [young and older participants over 65 years] and sex [males and females]) revealed that for SEEQ there was a significant main influence of age group (*F*(1/307) = 3.924, *p* = .048, *η*_p_^2^ = .013) with younger individuals reporting higher SEEQ (*M* = 76.38, *SD* = 11.04) compared to older individuals (*M* = 75.57, *SD* = 15.19), and there was a significant influence of sex (*F*(1/307) = 4.745, *p* = .030, *η*_p_^2^ = .015) with females reporting higher SEEQ (*M* = 77.06, *SD* = 10.96) compared to males (*M* = 74.45, *SD* = 15.82), as well as an interaction of age group × sex (*F*(1/307) = 54.150, *p* < .001, *η*_p_^2^ = .150) with old males reporting the lowest SEEQ, followed by young females, young males, and old females reporting the highest SEEQ (Table 4).

**TABLE 4:** Descriptive statistics regarding SEEQ in young and older adult groups according to sex

Age group	Sex	Mean	*SD*	95% CI		*N*
Young	Males	80.50	14.42	77.62	83.93	69
	Females	73.22	5.85	70.69	75.74	90
Old	Males	67.37	14.47	64.25	70.49	59
	Females	80.78	13.27	78.29	83.27	93

A two‐way ANOVA with two independent variables (age group [young and older participants over 65 years] and sex [males and females]) revealed that for physical attractiveness there was no significant main influence of age group (*F*(1/307) = 0.048, *p* = .827, *η*_p_^2^ < .001) and no significant influence of sex (*F*(1/307) = 0.133, *p* = .716, *η*_p_^2^ < .001), but there was a significant interaction of age group × sex (*F*(1/307) = 35.003, *p* < .001, *η*_p_^2^ = .102) with old males reporting the lowest, followed by young females, young males, and old females reporting the (unexpectedly and regardless of the gender and age stereotypes) highest physical attractiveness (Table 5).

**TABLE 5:** Descriptive statistics regarding physical attractiveness in young and older adult groups according to sex

Age group	Sex	Mean	*SD*	95% CI		*N*
Young	Males	80.39	14.82	77.73	83.04	69
	Females	73.21	3.34	70.87	75.53	90
Old	Males	72.45	8.61	69.58	75.32	59
	Females	80.58	13.06	78.29	82.86	93

A two‐way ANOVA with two independent variables (age group [young and older participants over 65 years] and sex [males and females]) revealed that for health ratings there was a significant main influence of age group (*F*(1/307) = 8.628, *p* = .004, *η*_p_^2^ = .027) with young adults showing higher health ratings (*M* = 76.10, *SD* = 11.13) compared to old adults (*M* = 72.93, *SD* = 14.75) and no significant influence of sex (*F*(1/307) = 0.069, *p* = .793, *η*_p_^2^ < .001), but there was a significant interaction of age group × sex (*F*(1/307) = 19.464, *p* < .001, *η*_p_^2^ = .060) with old males reporting the lowest health estimations, followed by young females, old females, and young males presenting the highest scores (Table 6).

**TABLE 6:** Descriptive statistics regarding health ratings in young and older adult groups according to sex

Age group	Sex	Mean	*SD*	95% CI		*N*
Young	Males	79.97	14.65	76.96	82.97	69
	Females	73.13	5.95	70.50	75.76	90
Old	Males	69.22	11.35	65.97	72.46	59
	Females	75.29	16.16	72.70	77.87	93

A two‐way ANOVA with two independent variables (age group [young and older participants over 65 years] and sex [males and females]) revealed that for optimism there was a significant main influence of age group (*F*(1/307) = 5.212, *p* = .023, *η*_p_^2^ = .017) with younger participants reporting more optimism (*M* = 3.41, *SD* = 1.12) compared to older participants (*M* = 3.13, *SD* = 1.07) and no significant influence of sex (*F*(1/307) = 0.972, *p* = .325, *η*_p_^2^ = .003), but there was a significant interaction of age group × sex (*F*(1/307) = 0.003, *p* = .959, *η*_p_^2^ < .001) with old males reporting the least optimism, followed by old females, young males, and young females reporting the highest optimism (Table 7).

**TABLE 7:** Descriptive statistics regarding optimism in young and older adult groups according to sex

Age group	Sex	Mean	*SD*	95% CI		*N*
Young	Males	3.34	1.12	3.08	3.60	69
	Females	3.46	1.13	3.23	3.69	90
Old	Males	3.05	1.02	2.76	3.33	59
	Females	3.18	1.10	2.95	3.40	93

A two‐way ANOVA with two independent variables (age group [young and older participants over 65 years] and sex [males and females]) revealed that for religiousness there was no significant main influence of age group (*F*(1/307) = 0.089, *p* = .766, *η*_p_^2^ < .001), no significant influence of sex (*F*(1/307) = 0.269, *p* = .604, *η*_p_^2^ = .001), and no significant interaction of age group × sex (*F*(1/307) = 0.356, *p* = .551, *η*_p_^2^ = .001) (Table 8).

**TABLE 8:** Descriptive statistics regarding religiousness in young and older adult groups according to sex

Age group	Sex	Mean	*SD*	95% CI		*N*
Young	Males	4.11	1.59	3.69	4.53	69
	Females	4.10	1.51	3.73	4.46	90
Old	Males	3.93	1.85	3.47	4.38	59
	Females	4.16	2.07	3.79	4.52	93

Two‐way ANOVAs with two independent variables (age group [young and older participants over 65 years] and sex [males and females]) revealed for all the administered neuropsychological tests only a main effect of age group, and no other influences of sex. More specifically, there were statistically significant main influences of age group with the older group scoring as expected lower, for the Corsi test (*F*(1/307) = 210.748, *p* < .001, *η*_p_^2^ = .407), for the Visual Patterns Test (*F*(1/307) = 390.547, *p* < .001, *η*_p_^2^ = .560), for the Digit Span Forward (*F*(1/307) = 11.842, *p* = .001, *η*_p_^2^ = .037), and for the Verbal Fluency Task (*F*(1/307) = 231.424, *p* < .001, *η*_p_^2^ = .430). No such significant influences of sex were found for the performance regarding Visual Patterns Test (*F*(1/307) = 0.001, *p* = .973, *η*_p_^2^ < .001), Corsi test (*F*(1/307) = 0.398, *p* = .528, *η*_p_^2^ = .001), Digit Span Forward (*F*(1/307) = 0.341, *p* = .559, *η*_p_^2^ = .001), and for the Verbal Fluency Task Forward (*F*(1/307) = 0.029, *p* = .864, *η*_p_^2^ < .001).

A stepwise regression was conducted with SEI as dependent variable and the above correlates as independent variables (*R* ^2^ = .703, *F*(4, 271) = 160.077, *p* < .001), revealing four significant predictors: sex (*B* = −4.326, *t* = 4.793, *p* < .001, 95% confidence interval [CI] [−6.103, −2.549]), physical attractiveness (*B* = 0.752, *t* = 9.284, *p* < .001, 95% CI [0.593, 0.912]), SEEQ (*B* = 0.164, *t* = 2.266, *p* = .024, 95% CI [0.021, 0.306]), and age (*B* = 0.072, *t* = 3.734, *p* < .001, 95% CI [0.034, 0.110]), while no other variables were significant.

## 4 DISCUSSION

Although so far findings support that males of all ages tend to estimate their general intelligence about 5–15 IQ points higher than do females, usually around 1 *SD* above the norm (Furnham, 2017; Furnham & Grover, 2020), this is not the case for this Greek sample of older adults. Males had higher SEI only for the group of young adults, while the reverse was found for older adults as in this group females reported higher SEI. In addition to that, EI followed the same pattern of self‐estimations for the interaction of sex and age groups. The view of an “MHFN bias” was not fully confirmed as men in the present study awarded themselves significantly higher estimates for overall and EI, only when they belonged to the group of young adults (Furnham et al., 1999). This sex and age interaction could be explained by affect differences as measured by negative PANAS, which was higher for older men.

Thus, age seems to be of utmost importance when SEI is examined. So far, older adults are not represented in relevant research as convenience samples are usually used (Cherubini & Gasperini, 2017). According to the current findings, the age dimension plays a vital role for SEI and SEEQ in persons aged 65 years or over, so although it has been neglected in relevant literature, future research attempts should consider it as an equally important variable as sex. Although it is difficult to disentangle what may be historical cohort effects (e.g., lack of available access to education) from what might be a biopsychosocial effect of cognitive aging and the understandable downward estimation of one's intelligence, this study points to a new question that needs to be elucidated in future research: Are the findings due to cross‐cultural differences, is it a historical cohort effect related to access to education (better and longer educated younger adults), or do the frequently observed sex differences in SEI not generalize to older populations?

When objective cognitive tests of WM are used (such as the Visual Patterns Test), it is interesting that SEI does not correlate with them, something that is an unexpected finding, given that higher objective performance should support estimates of higher IQ. A real strength of this study is that it is backed up by neuropsychological measurements. Thus, for the first time we are able to rule out the presence of neurological impairment in the study or that SEI is an accurate reflection of the difficulties the participants encountered in the neuropsychological battery. Although direct cognitive performance was assessed (as indicated by the four neuropsychological tests), the objective psychometrically measured cognitive ability does not shape self‐perceptions such as SEI, a finding that is also reported in Greek older adults with mild cognitive impairments and healthy Greek older adults (Fragkiadaki et al., 2016; Giannouli & Tsolaki, 2022b).

Another point that needs to be taken into consideration is physical attractiveness, which differentiates age and sex groups, as it is highest for old females, followed by young males, young females, and old males. Physical attractiveness has a high correlation with SEI. Also, physical attractiveness may act as a proxy for general self‐esteem, and it has been supported that self‐esteem is a strong component of the SEI (Reilly et al., 2022). Another point that is remarkable is that there was no correlation between SEI and optimism, a finding that allows us to support that the participants just do not have an optimistic bias toward overestimation. This is in contrast with studies supporting a relationship between optimistic bias, narcissism, and subjectively assessed intelligence (Zajenkowski & Gignac, 2018).

As in a previous study (Furnham & Grover, 2020), demographic variables (sex, age, education), self‐ratings (attractiveness, EQ, and health), and non/antiscientific beliefs (regarding religion that has been found to be of utmost importance for Greek older adults; Giannouli & Giannoulis, 2021a, 2021b; Giannoulis & Giannouli, 2020b) were included. Furthermore, as this is the first time that such variables are simultaneously examined in Greece in an extended sample of individuals with varying demographic characteristics, it is worth mentioning the interaction effects for all these self‐ratings, as the effect of the sex factor depends on the other factor, which in our case is the age group for SEI, SEEQ, physical attractiveness, and health ratings.

Although high levels of religiousness in Greece are reported in prior research for the old age group (Van Herreweghe & Van Lancker, 2019), both younger and older adults of both sexes in this sample had moderate levels of self‐reported religiousness, possibly due to the COVID‐19 crisis‐imposed restrictions that changed relevant religious behaviors, such as church attendance and public worship (Giannoulis & Giannouli, 2021). Findings revealed that perceived religiousness positively correlated with SEI, SEEQ, and health ratings, but no direct relationship to optimism has been found in contrast to assumed links. The fact that perceived religiousness may not play a role in motivating positive attitudes, including optimism, could be due to the severe health and financial conditions in Greece at the time of assessment of the participants.

This study was not only a replication study, as creativity was also assessed taking the form of attitudes and values. Another novelty of this study was the neuropsychological assessment of WM. For SEI, four were the significant predictors, namely, age (older), sex (male), physical attractiveness, and SEEQ, while no other variables were found to be significant. Among them, self‐estimated creativity negatively correlates with SEI only for the group of younger adults, something that could be explained by a different perception of these two psychological constructs (intelligence, creativity) as unrelated and distinct (Furnham et al., 2008).

This may have important implications for psychiatric and neuropsychological assessment as women may show overconfidence in their cognitive abilities, and this may drive them to show less willingness to get assessed based on the higher SEI that older women report or to report distorted data for themselves and their cognitive self‐image, but also there might be implications for the everyday living of community‐dwelling older women and men, especially in shared environments by both sexes. Although there was a hypothesis that objective WM test performance (concerning visual and verbal aspects of WM) would correlate with SEI, this was not the case. Another interesting neuropsychological finding that should be mentioned here is that although sex is expected to influence verbal capacity performance (e.g., Verbal Fluency Task), sex did not differentiate women and men regarding their phonemic fluency, something that reaffirms that in the Greek population sex contributes only to total word production on the semantic task and that sex differences in specific categories may reflect and be explained by sociocultural factors (Kosmidis et al., 2004).

A point that could be considered as a limitation of this study is that “objective” (i.e., psychometric) intelligence was not directly tested due to the fact that a lengthy testing session is not appropriate for older adults, but also the administration of all of the supplemental subtests to young adults has been criticized for having long administration times and causing fatigue (Greene, 2000), and due to copyright–proprietary issues for the only IQ test in use in Greece (current version of WAIS). However, by including a neuropsychological battery such as verbal fluency and Corsi blocks, we can rule out these results being driven by perceived or actual neuropsychological impairment as a result of aging.

Another limitation is the debate regarding WM and the appropriateness of the included measures (digit forward, visual patterns, and backward Corsi block), which could be better classified as measuring something other than WM, and be more indicative of STM than WM (Shao et al., 2014), while verbal fluency measures are supported to be primarily linked to executive functions (Amunts et al., 2020). A third limitation of this research may be the fact that given that the questions on the 0–100 scale for the self‐estimations were presented the one after the other, many participants may have responded automatically with the same or similar reports, without making conscious estimations. Additionally, all participants were Greek Orthodox Christians, so the role of religiousness should be examined through the prism of one single religion. Another point is the fact that overall SEI was measured, and not multiple intelligences following Gardner's theory, while prior test experience was homogeneously present for all participants and could not be included in the analyses as a possible influence (Furnham et al., 2009). Of course, neuropsychological test scores revealed age differences, something that is generally expected in neuropsychological research regardless of the examined cognitive function (Lezak et al., 2012), given that normal ageing degrades the information processed, thus impairing cognitive processing (Schneider & Pichora‐Fuller, 2000).

Future research should extend the current findings with the simultaneous examination of personality factors, apart from the state affect factors. Additionally, creativity could be examined in a more detailed way, as the Creative Attitudes and Values may not reflect the “actual” creativity but the attitudes and values that shape involvement in creative behaviors and activities. One more point is that sex should not be confused with gender, which refers to the socially constructed characteristics of women and men, and not to the biological sex, and thus two these two concepts should be examined and inserted into the analyses, separately (Reilly et al., 2022).

## CONFLICT OF INTEREST

The author declares no conflict of interest.

### PEER REVIEW

The peer review history for this article is available at https://publons.com/publon/10.1002/brb3.2857.

## Data Availability

Data available on request due to privacy restrictions.
